# The prevalence of coronary anomalies in a single center of Korea: origination, course, and termination anomalies of aberrant coronary arteries detected by ECG-gated cardiac MDCT

**DOI:** 10.1186/1471-2261-14-48

**Published:** 2014-04-12

**Authors:** June Namgung, Jeong A Kim

**Affiliations:** 1 Division of Cardiology, Department of Internal Medicine, Vision 21 Cardiac & Vascular Center, Ilsan Paik Hospital, Inje University College of Medicine, Goyang, 170 Juhwa-ro, Ilsanseo-gu, Goyang-si, Gyeonggi-do 411-706, Republic of Korea; 2 Department of Radiology, Ilsan Paik Hospital, Inje University College of Medicine, Goyang, Republic of Korea

**Keywords:** Coronary vessel anomalies, Multidetector computed tomography, Prevalence

## Abstract

**Background:**

Coronary anomalies are rare congenital abnormalities often found incidentally on conventional coronary angiography (CCA) or coronary CT angiography (CTA). They may result in various clinical outcomes. CCA is invasive and not able to demonstrate all coronary anomalies in detail, especially those with complex courses. Multidetector computed tomography (MDCT) enables visualization of the origin and course of coronary arteries. The objective of this study was to investigate the prevalence of origin and termination coronary artery anomalies and the course of these anomalies in patients in a single center in Korea.

**Methods:**

To diagnose coronary anomalies, the angiographic data of 8,864 consecutive patients undergoing 64- or 320-MDCT from September 2005 to November 2011 were analyzed retrospectively.

**Results:**

Among the 8,864 patients, 103 (1.16%) had coronary anomalies. Ninety (87.4%) patients had origin and distribution anomalies, and 13 (12.6%) patients had a coronary artery fistula. The most common anomaly (41, 39.8%) was an anomalous origin of the right coronary artery (RCA). Of these, three patients received a coronary artery bypass graft.

**Conclusions:**

The prevalence of coronary anomalies in a single center of Korea was 1.16%. The incidence and patterns of coronary artery anomalies in our patient population were similar to those of previous studies.

## Background

The prevalence of coronary artery anomalies is reported to be approximately 0.3% to 2% of the general population [1-11]. Most of these anomalies are asymptomatic during life, and the prognosis is good. However, some of these anomalies are associated with syncope, ischemic heart disease, and sudden death [12-17]. These anomalies are detected as incidental findings during coronary angiography or at autopsy. Conventional coronary angiography (CCA) is widely available and considered the gold standard diagnostic tool in coronary anatomy. However, CCA is invasive and not able to demonstrate all coronary anomalies in detail, especially complex ones with anomalous courses. Recent advances in computed tomography (CT) techniques, such as multidetector computed tomography (MDCT) scanners, allow noninvasive visualization of the origin and course of coronary arteries. The aim of this study was to evaluate the prevalence of coronary anomalies in consecutive patients who underwent MDCT coronary angiography at Ilsan Paik Hospital, Inje University College of Medicine in Korea.

## Methods

### Patients

This retrospective study consisted of 8864 patients (5110 men, 3754 women; mean age 62 ± 13 years) who underwent ECG-gated MDCT from September 2005 to November 2011 at Ilsan Paik Hospital, Inje University College of Medicine. The database consisted of all consecutive patients undergoing cardiac 3D CT of the coronary artery, which is one of our hospital’s radiological imaging tests. Other CT examinations of our hospital’s radiological studies containing chest portion (such as chest 3D CT, 3D CT angio thracoabdominal aorta, and thoracic aorta for cardioembolic source work-up) were excluded. The indications for performing coronary CT angiography (CCTA) were chest pain, evaluation of syncope, angina pectoris, evaluation of cardiomyopathy, preoperative evaluation for noncoronary surgery, screening for coronary artery disease, and determination of the patency of bypass grafts or stents. Exclusion criteria for CCTA were uncontrollable arrhythmia or a resting heart rate over 80 bpm, any previous serious allergic reaction to the contrast medium, pregnancy, renal failure (serum creatinine > 3.0 mg/dL) and respiratory difficulties (physician’s discretion). CCTA was conducted using a 64-MDCT in 7,054 patients and 320-MDCT in 1,810 patients. We selected patients with coronary anomalies and reviewed the origin, course, and termination of their coronary arteries. We excluded myocardial bridges in the category of coronary anomalies because of the lack of clarity regarding its definition [18,19]. This retrospective study was approved by the institutional review board of Inje University Ilsan Paik Hospital (IRB No. IB-3-1402-008). The need for informed consent was waived by the board.

### Scan protocol and image acquisition

Prior to CCTA, all patients with a baseline heart rate of >65 beats/min received 10–30 mg of esmolol (Jeil Pharm, Seoul, Korea) intravenously. Nitroglycerin (0.6 mg) was given to all the patients sublingually 1 minute before contrast injection. The patients’ heart rates, ECG, and blood pressure were checked throughout the procedure.

Scanning was performed using a 64-MDCT scanner (Aquilion 64, Toshiba Medical Systems, Otawara, Japan) or a 320-MDCT scanner (Aquilion ONE, Toshiba Medical Systems). After the topograms, an ECG-gated sequential precontrast scan was performed with the following parameters: 3 mm of collimation, 0.4 seconds of rotation time with half exposure, 120 kV, 180 mm field of view, and 300 mA in the 64-MDCT scanner; 320 × 0.5 mm collimation, 350 ms gantry rotation time, and 175 ms temporal resolution in the 320-MDCT scanner. The obtained images were used for the determination of scan range and positioning of the heart in the center of the field of view. In the contrast-enhanced coronary CT scan, a retrospective ECG-gated spiral scan was obtained with 0.5 mm of collimated section thickness (64 × 0.5 mm collimation), a pitch of 0.204 to 0.224 (automatically selected depending on the patient’s pulse rate), a rotation time of 0.4 seconds, an 180 mm field of view, a tube voltage of 120 kV, and a tube current of 150–500 mA. The tube current was modulated individually using the standard deviation (SD) of the CT numbers obtained during the precontrast scanning at the level of the left atrium. The automatic triggering system was activated by the presence of 140 to 160 Hounsfield units (HU) at the aortic root after the injection of 60 to 80 mL (scaled according to body weight) of nonionic contrast (Iomeron 400, Bracco, Milan, Italy) at a rate of 4.5 mL/s, followed by 30 mL of normal saline.

### Postprocessing and image analysis

Retrospective ECG-gated reconstructions were obtained from 70%, 75%, and 80% of the R-R interval. Data from the gated protocol were sent to the image processing workstation (Rapidia; Infinitt, Seoul, Korea). Routine curved multiplanar reconstructed images and 3D volume rendered images were evaluated in all patients. The CT scans were reviewed by one radiologist and one cardiologist, and decisions were reached by consensus.

## Results

Among the 8,864 patients, 103 (1.16%) had coronary anomalies in this retrospective study. Their mean age was 60 ± 13.7 years (age range: 23 to 88, male:female ratio = 67:36). The baseline clinical characteristics of the patients are shown in Table 1. Ninety (87.4%) patients had origin and distribution anomalies, and 13 (12.6%) had a coronary artery fistula (Table 2).

**Table 1:** Baseline clinical characteristics of the 103 patients with coronary anomalies


Age, years (range)	59.7 ± 14 (23–88)
Gender	M:F = 67:36
Presenting symptom	
Typical chest pain	44 (42.7%)
Atypical chest pain	31 (30.1%)
No chest pain	
Dyspnea	8 (7.8%)
Syncope	9 (8.7%)
Palpitation	5 (4.9%)
Free	6 (5.8%)
Risk factors	
Diabetes	23 (22%)
Hypertension	52 (52%)
Dyslipidemia	41 (40%)
Current smoker	13 (13%)
Cerebrovascular accident	1 (1%)
Underlying cardiac problems	
Previous myocardial infarction	2 (1.9%)
Previous coronary intervention	1 (1.0%)
Dilated cardiomyopathy	2 (1.9%)
Detector number of MDCT	
64 row	73 (71%)
320 row	30 (29%)

**Table 2:** Prevalence of coronary anomalies

**Coronary anomaly**	**Number of patients (*n*** **= 103, 1.16%)**	**Anomaly incidence among 8864 patients (%)**	**Constituent ratio among 103 cases (%)**
Origin and course of anomalies			
Anomalous origin from opposite sinus, with anomalous course			
RCA arising from the left anterior sinus, with an interarterial course	41	0.463	39.8
LCX arising from the right anterior sinus or RCA, with a retroaortic course	5	0.056	4.9
LMCA arising from the right anterior sinus, with an interarterial course	1	0.011	1.0
Multiple ostia			
Absent left main trunk (split origin of LCA)	10	0.112	9.7
RCA and conus branch arising separately	7	0.080	6.8
Single coronary ostium	1	0.011	1.0
Anomalous location of coronary ostium in the correct coronary sinus			
High takeoff			
RCA	9	0.102	8.7
LMCA	6	0.070	5.8
RCA and LMCA	2	0.023	1.9
Commissural	4	0.045	3.9
Duplication of arteries (type IV dual LAD)	4	0.045	3.9
Coronary termination anomalies (coronary artery fistulas)			
RCA-PA	1	0.011	1.0
RCA-RA	1	0.011	1.0
LAD-PA	6	0.070	5.8
LAD-RCA	1	0.011	1.0
LCX-bronchial artery	1	0.011	1.0
RCA-PA and LAD-PA	3	0.034	2.9

**Figure 1:**
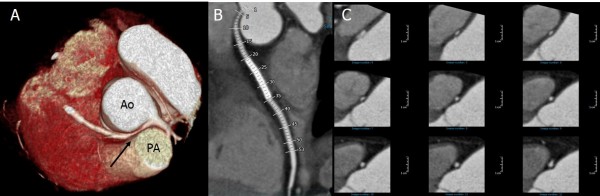
**Anomalous origin of the right coronary artery (RCA) originating from the left coronary sinus of Valsalva (LSV).** 3D volume-rendered coronary image **(A)** and curved, multiplanar reconstruction image **(B)** showing the anomalous origin of the right coronary artery (black arrow) from the LCS, and courses between the aorta (Ao) and the pulmonary artery (PA). Cross-sectional image **(C)** showing the proximal RCA, with narrowing and lateral compression resulting in an ovoid lumen.

Origin anomalies were the most common abnormality, with the coronary artery originating in the opposite coronary sinus. Of these anomalies, the most common type was a right coronary artery (RCA) originating from the left sinus of Valsalva (LSV) (41 patients, 39.8%) (Figure 1). All these anomalies passed between the aortic root and the pulmonary artery (interarterial course). Thirty-five of these 41 patients showed significant luminal narrowing of the opening of the RCA. Of these, two patients underwent a coronary artery bypass graft, and one patient underwent an unlooping procedure.

**Figure 2:**
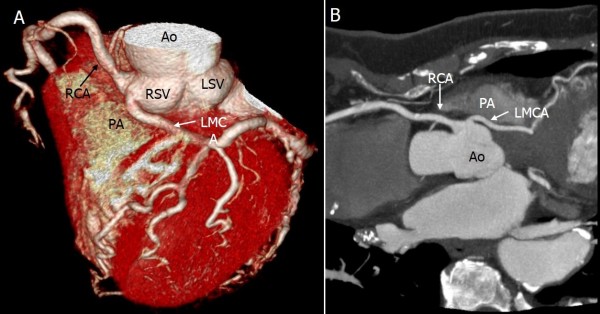
**Origin and course of the anomalies of the left main coronary artery (LMCA) from the right sinus of Valsalva (RSV).** Volume rendering image shows the LMCA arising from the RSV **(A)**. It depicts the long LMCA branching into the left circumflex artery at the proximal interventricular groove and the RCA arising normally from the RSV. The curved multiplanar reconstruction image **(B)** shows the opening of the LMCA, acute angle take-off of the RSV from the aorta, and the interarterial course between the ascending aorta and the pulmonary artery (PA).

**Figure 3:**
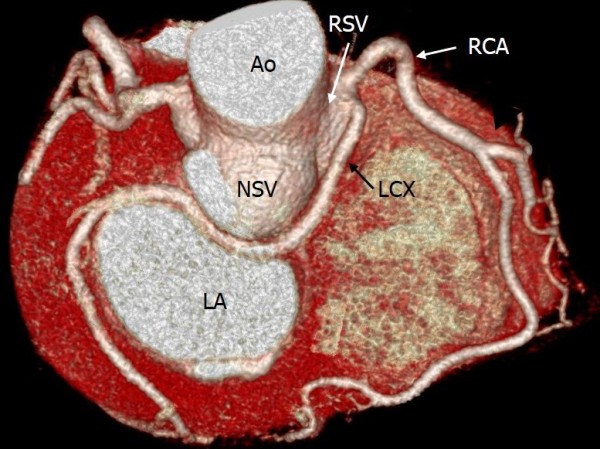
**Anomalous origin of the left circumflex artery (LCX) from the right sinus of Valsalva (RSV).** Volume rendering image shows the LCX arising from the RSV **(A)** or the right coronary artery **(B)**, and travelling between the aorta and the left atrium (retroaortic course).

The left main coronary artery originated from the right sinus of Valsalva (RSV) in one case (Figure 2). This case also showed an interarterial course. In five patients, the left circumflex artery (LCX) arose from the RSV (3 patients, 2.9%) or the RCA (2 patients, 1.9%). In all these cases, the artery passed behind the aortic root (retroaortic course) (Figure 3).

**Figure 4:**
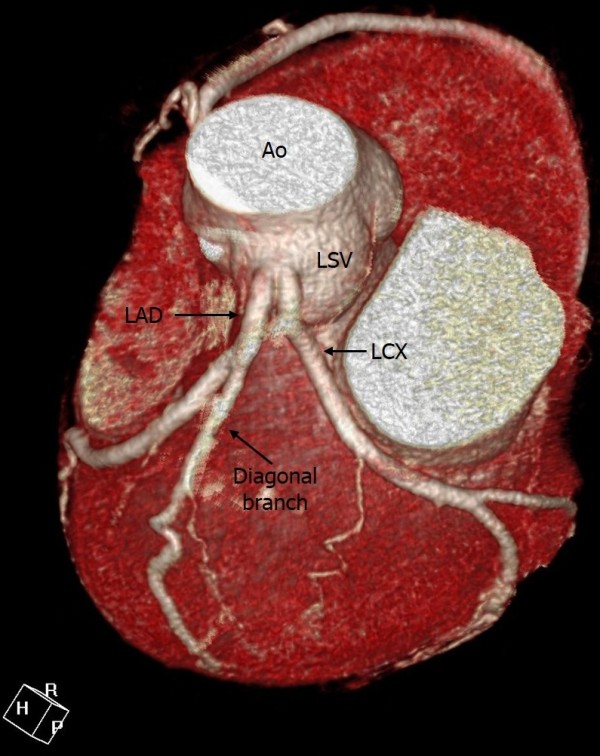
**Absent left main trunk (split origin of left coronary artery).** 3D volume rendering image shows absent left main coronary artery (LMCA) and the separate origin of the left anterior descending coronary artery (LAD) and left circumflex artery (LCX).

**Figure 5:**
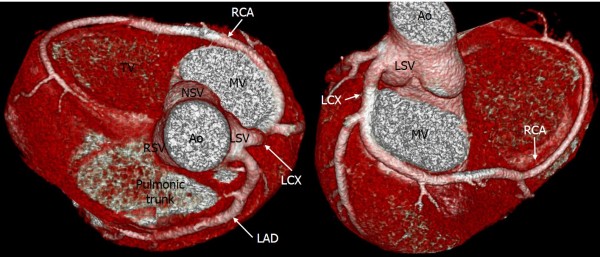
**Volume rendering image of single coronary ostium in the left sinus of Valsalva (LSV).** The dilated left main coronary artery (LMCA) divided into the left anterior descending coronary artery (LAD) and the left circumflex artery (LCX). The LCS then coursed in the left atrioventricular groove and continued to the posterior atrioventricular groove where it occupied the anatomic position normally occupied by the right coronary artery (RCA).

Ten (9.7%) patients had no left main coronary artery (LMCA) and a separate ostium of the left anterior descending coronary artery (LAD) and LCX from the LSV (Figure 4). In seven patients (6.8%), the RCA and conus branch showed a separate origin from the RSV. One patient had a single coronary artery arising from the LSV. The LMCA divided normally into the LAD and the LCX. This patient’s RCA existed as a continuation of the distal LCX, with a retroaortic course (Figure 5).

**Figure 6:**
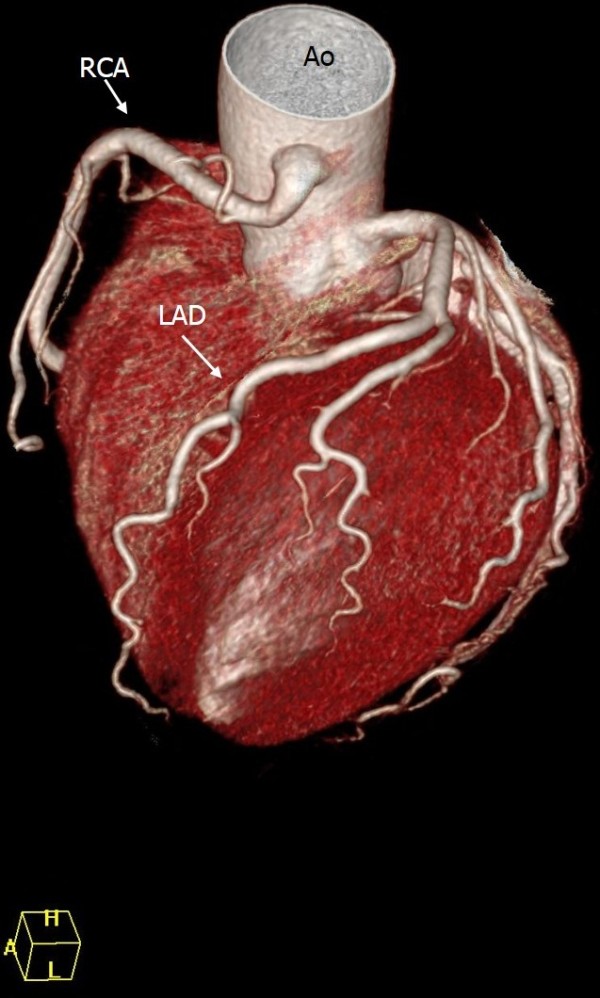
**High takeoff of the right coronary artery (RCA).** The volume rendering image shows the origin of the RCA from the anterior surface of the ascending aorta approximately 2 cm above the sinotubular junction. The first portion of the proximal RCA shows a hockey-stick type of configuration, with an acute-angle bend on its main stem, before returning to its regular course in the right atrioventricular groove.

We found 17 (16.5%) high-takeoff coronary arteries. Five RCAs coursed between the aorta and the pulmonary artery, resulting in compression and narrowing of the RCA lumen (Figure 6); and six RCAs and seven coronary arteries had a normal angulation and course.

**Figure 7:**
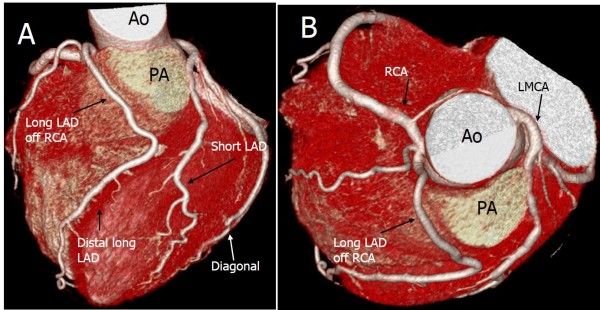
**Duplication of the left anterior descending coronary artery (LAD). A**: 3D reconstruction, showing the course of the long LAD from its origin in the right coronary artery (RCA), then passing anterior to the pulmonary artery (PA) and following a course to the anterior interventricular groove where it follows the course of a traditional distal LAD. The short LAD courses and terminates in the anterior interventricular sulcus, without reaching the apex. **B**: 3D reconstruction showing the left main coronary artery (LMCA) and its short LAD branching the diagonal branches.

Duplication of the LAD was found in four patients. In all these patients, the long course of the LAD originated from the RCA. The LAD had numerous septal branches and travelled through the interventricular groove before terminating at the cardiac apex. One LAD originated from the normal site and travelled through the anterior cardiac border, following a similar course to the diagonal branches (Figure 7).

Coronary artery fistulas were detected in 13 (12.6%) patients. Among these patients, fistulas arising from the LAD to the pulmonary artery were the most common.

## Discussion

The prevalence of coronary artery anomalies in our patients was 1.16%. In the largest study (*n* = 26,595) conducted by the Cleveland Clinic Foundation in North America in 1990, the incidence was 1.3% (*n* = 1,686). The findings in our study are similar to those described in the literature (Table 3). Thereafter, several studies conducted in different countries reported similar results. To the best of our knowledge, this is the first study to investigate the prevalence of coronary anomalies in the general Korean population.

**Table 3:** Prevalence of coronary anomalies in previous published studies

**Author/year of publication**	**Prevalence (%)**	**Most common coronary anomaly**	**Imaging modality**	**Country**
Yamanaka et al. 1990 [1]	1.30 (1,686 of 126,595)	Absent LMCA with separate origin of LAD and LCX	CAG	USA
Kaku et al. 1996 [2]	0.31 (56 of 17,731)	Anomalous origin of RCA from LSV	CAG	Japan
Kardos et al. 1997 [3]	1.34 (103 of 7,694)	Absent LMCA with separate origin of LAD and LCX	CAG	Central Europe
Garg et al. 2000 [4]	0.95 (39 of 4,100	Anomalous origin of RCA from LSV/NAS	CAG	India
Yildiz et al. 2010 [5]	0.90 (112 of 12,457)	Absent LMCA with separate origin of LAD and LCX	CAG	Turkey
Erol et al. 2011 [6]	1.96 (53 of 2,096)	Absent LMCA with separate origin of LAD and LCX/Origin of RCA from LSV	64MDCT	Turkey
Fujimoto et al. 2011 [7]	1.52 (89 of 5,869)	Anomalous origin of RCA from LSV	64MDCT	Japan
Sivri et al. 2012 [8]	0.74 (95 of 12,814)	LCX arising from RSV or RCA	CAG	Turkey
Sohrabi et al. 2012 [9]	1.30 (79 of 6,065)	Absent LMCA with separate origin of LAD and LCX	CAG	Iran
Xu et al. 2012 [10]	1.02 (124 of 12,415)	Anomalous origin of RCA from LSV	Dual-source CTCA	China
Yukel et al. 2013 [11]	0.29 (48 of 16,573)	Anomalous origin of LCX from RCA/RSV	CAG	Turkey

The etiology of coronary anomalies is uncertain. There is no definite inheritance pattern and no sex predominance. In a previous study, the most frequent coronary anomaly was the origin of the RCA from the LSV and an absent LMCA [2,4,7,10], with a separate origin of the LAD and LCX [1,3,5,6,9]. In our population, the origin of the RCA from the LSV was also the most common coronary anomaly (39.8%, 41 of 103 patients). This is a clinically significant anomaly because the interarterial course between the pulmonary aorta and the aorta and the compression of the RCA ostium may induce myocardial ischemia or sudden death. Anomalous origins of coronary arteries, where the artery crosses over to the opposite sinus, show four patterns: (1) an anterior course anterior to the pulmonary trunk or the right ventricular outflow tract, (2) an interarterial course between the pulmonary artery and the aorta, (3) a septal course through the interventricular septum, and (4) a retroaortic course posteriorly between the aortic root and the left atrium [20]. Of these, the interarterial course is clinically malignant because it is strongly associated with sudden death, myocardial ischemia, congestive heart failure, and endocarditis. The exact pathophysiological mechanisms of myocardial ischemia have not been determined. Cheitlin et al. [12] reported that a left coronary artery (LCA) arising as a single or double vessel from the anterior sinus of Valsalva, where the LCA passed leftward and posteriorly between the aorta and the pulmonary artery, is associated with sudden death. The assumed mechanism of sudden death is ostial closure between the aorta and pulmonary artery and the squeezing of the ostium during exercise, with sudden interference in coronary arterial flow [21,22]. In such cases, complicated cardiac surgery or interventions are needed. Three of these patients of anomalous origin of the RCA in our hospital underwent coronary bypass surgery or coronary unroofing surgery. To evaluate the risk posed by a coronary anomaly, it is very important to discriminate an interarterial course from other courses. CCA is usually unable to provide information on the complex anatomy of coronary anomalies. ECG-gated MDCT is not only a noninvasive diagnostic tool but also a precise instrument for delineating the exact origin and course of coronary anomalies using 3D reconstruction. In planning surgery or coronary angioplasty for coronary anomalies, MDCT provides a more accurate picture of the origin and course of the coronary vessels than CCA. The main disadvantage of MDCT is the high exposure to radiation. Compared with 64-MDCT, 320-MDCT has better temporal resolution, with faster image acquisition and a shorter scanning time, thereby decreasing the radiation exposure of patients. The 64-slice CT may expose patients to more radiation, which might be unacceptable when screening young symptomatic individuals with suspected coronary anomalies [23]. Therefore, the new 320-slice MDCT has the potential to become the technique of choice for noninvasive diagnosis of coronary anomalies.

In previous studies, a separate origin of the LAD and LCX arteries from the LSV was the most common anomaly, occurring in 30–60% of cases. In the present study, the incidence was 9.7% (10 of 103). A common cause of multiple ostia is the conus branch arising directly from the aorta rather than the proximal RCA. This anomaly was seen in seven (1.9%) patients in the present study.

A single coronary ostium is extremely rare. Shirani et al. [24] classified a solitary coronary ostium into 20 categories according to the location of this anomaly. In our study, only one patient had a solitary coronary ostium. A solitary ostium in the LSV normally occurs in the LAD and LCX, and the RCA exists as a continuation of the distal LCX, coursing to the posterior and right atrioventricular groove.

A coronary anomaly with a high takeoff or ectopic origin refers to a left or right coronary ostium, which arises more than 0.5 cm above the sinotubular junction rather than at the aortic sinus [25,26]. This is a hemodynamically benign coronary anomaly and usually considered a normal variant, but it may cause difficulties in cannulation during coronary angiography and coronary artery bypass surgery [27].

Dual LAD is a rare coronary anomaly. Spindola-Franco et al. [28] described four variations of dual LAD. In type IV, the long LAD arises from the RCA and enters the distal anterior interventricular groove, whereas the short LAD originates from the LCA and ends high in the anterior interventricular groove. Four patients in our study had a type IV coronary anomaly according to Spindola-Franco et al.’s classification. However, the anomaly differed somewhat from their description. The long LAD arising from the RCA passed anterior to the pulmonary artery and travelled anterior interventricular grooves where it followed the course of a traditional distal LAD. Another long LAD originating from the LCA coursed and terminated in the anterior left ventricular wall, without reaching the apex.

Coronary artery fistulas refer to abnormal connections between one or more of the coronary arteries and the heart chamber or another blood vessel, such as the pulmonary artery, the coronary sinus, or the superior vena cava. Most coronary artery fistulas are small, do not cause any symptoms [29], and are clinically undetectable until echocardiography or coronary arteriography is performed for an unrelated cause. Small fistulas usually do not cause any hemodynamic compromise. However, larger fistulas can give rise to the coronary artery steal phenomenon, which leads to ischemia of the segment of the myocardium perfused by the coronary artery [30]. In our study, the most common fistula was a connection between the coronary artery and the pulmonary artery, and none of the fistulas resulted in clinical problems.

### Study limitations

Due to the retrospective design of this study, some important clinical characteristics may not have been recorded. Our study is a descriptive one, and it was not possible to compare the findings with those obtained using other modalities. We excluded myocardial bridges and other minor coronary anomalies, such as coronary ectasia or aneurysms. Myocardial bridges were excluded because there are no clear diagnostic criteria and no unified classification of the multiple variations.

## Conclusions

The prevalence of coronary anomalies in this study was similar to that of previous studies. Coronary anomalies are rare. However, as some types of coronary anomalies are clinically significant and sometimes life threatening, it important to recognize that they are detected in angiographic images. ECG-gated MDCT is a noninvasive technique, with high temporal and spatial resolution. The 3D images obtained with MDCT can be used to provide accurate angiographic information on the origin, course, and termination of coronary anomalies, which cannot be visualized with conventional coronary angiography. Understanding coronary anomalies can aid the physician in treatment planning.

## Abbreviations

CCA: Conventional coronary angiography; CCTA: Coronary CT angiography; LAD: Left anterior descending coronary artery; LCX: Left circumflex artery; LMCA: Left main coronary artery, LSV, left sinus of Valsalva; MDCT: Multidetector computed tomography; RCA: Right coronary artery; RSV: Right sinus of Valsalva.

## Competing interests

The authors declared that they have no competing interests.

## Authors’ contributions

JN: conception and design, acquisition, analysis, and interpretation of data, interpretation of CT images, draft of the manuscript; AK: interpretation of CT images. All authors read and approved the final manuscript.

## Pre-publication history

The pre-publication history for this paper can be accessed here:

http://www.biomedcentral.com/1471-2261/14/48/prepub
